# Digitising historical sea level records in the Thames Estuary, UK

**DOI:** 10.1038/s41597-022-01223-7

**Published:** 2022-04-12

**Authors:** Addina Inayatillah, Ivan D. Haigh, James H. Brand, Katy Francis, Alex Mortley, Matthew Durrant, Laura Fantuzzi, Elizabeth Palmer, Callum Miller, Peter Hogarth

**Affiliations:** 1 School of Ocean and Earth Science, National Oceanography Centre Southampton, University of Southampton, Waterfront Campus, European Way, Southampton, SO14 3ZH UK; 2 Environment Agency, 3rd Floor, Seacole Building, 2 Marsham Street, London, SW1P 4DF UK; 3 Port of London Authority, London River House, Royal Pier Road, Gravesend, Kent DA12 2BG UK; 4 National Oceanography Centre, Joseph Proudman Building, 6 Brownlow Street, Liverpool, L3 5DA UK

**Keywords:** Physical oceanography, Natural hazards

## Abstract

London is one of the world’s most important coastal cities and is located around the Thames Estuary, United Kingdom (UK). Quantifying changes in sea levels in the Thames Estuary over the 20^th^ century and early part of the 21^st^ century is vital to inform future management of flood risk in London. However, there are currently relatively few long, digital records of sea level available in the Thames. Here we present a new extensive sea level dataset that we have digitised from historical hand-written tabulated ledgers of high and low water, from the Port of London Authority (PLA). We captured 463 years of data, from across 15 tide gauge sites, for the period 1911 to 1995. When these historical datasets are combined with digital records available from the PLA since 1995, the sea level time-series span the 111-year period from 1911 to 2021. This new dataset will be of great importance for ongoing monitoring of mean sea-level rise, and changes in tidal range and extreme sea levels in the Thames Estuary.

## Abstract (summary)

**Table:** 

Measurement(s)	Sea level
Technology Type(s)	tide gauge
Factor Type(s)	high water
Sample Characteristic - Environment	Estuary
Sample Characteristic - Location	Thames Estuary

## Background & Summary

Understanding changes in mean sea-level is of the utmost importance, as it is a key indicator of climate change and it affects the livelihoods of hundreds of millions of people living in the world’s coastal regions^1^. Rising mean sea levels threaten low-lying coastal areas in many ways, including increasing extreme sea-levels^2^ which can give rise to serious coastal flooding and erosion^3^. Coastal cities are where the largest increase in losses from extreme sea level events can be expected. Hallegate *et al*.^4^ quantified present and future coastal flood losses in 136 of the largest coastal cities, worldwide. They estimated that the population exposed to flooding risk may grow by more than a factor of three in these cities due to the combined effects of mean sea-level rise, land subsidence, population growth and urbanization, with asset exposure increasing to more than ten times current levels. Quantifying changes in both mean and extreme sea levels over the 20^th^ century and early part of the 21^st^ century, at global, regional, and the local level of a particular city is therefore vital to inform future coastal management and planning decisions^5^.

London, located on the Thames Estuary in the UK, is one of the world’s most important coastal cities, and the largest city in northern Europe. It has been estimated that up to 1.42 million people and £321 billion worth of residential property in London, Kent and Essex would currently be exposed to a 0.5% annual probability of tidal flooding without the Thames Barrier and many hundreds of kilometres of associated defences^6^. Furthermore, within the Thames tidal flood zone there are also 496 education facilities, 711 healthcare facilities, 4 world heritage sites, and designated habitat sites, as well as critical energy, transport and water infrastructure. This includes the Port of Tilbury and London Gateway Port, the Blackwall Tunnel and Dartford Crossing, 167 km of rail routes, 116 train or tube stations, over 2400 km of paved roads, and 9 power stations.

Recognising the need to move from reactive to proactive flood risk management, to manage rising mean sea-levels and the aging defence network, the UK Environment Agency (EA) developed the Thames Estuary 2100 Plan^5^ (TE2100) to provide strategic direction for the continued management of flood risk in the Thames Estuary through to the end of the 21^st^ century and beyond. The Plan was instrumental in introducing a novel, cost effective approach to manage increasing flood risk by defining adaptation pathways, which embraced uncertainty in future changes in climate change^7^. A possible ‘route’ of ‘no regrets’ defence upgrades could be initially followed, with decisions on the most appropriate future pathway, e.g. raising the existing Thames Barrier or constructing a new barrier, being made later as understanding of the rate of climate change improves. The timing of defence upgrades, e.g. raising defences downstream of the Thames Barrier, and decision dates for future pathways can be brought forward if mean and extreme sea levels are found to be increasing faster than predicted.

For an adaptive approach to be effective, key indicators of change must be monitored and reviewed regularly. In the TE2100 Plan, 10 key indicators of change are being monitored, the first two of which are changes in mean and extreme sea levels. A formal review of these indicators is undertaken every 5 years (the first review was completed in 2016 and the second was completed in 2021^6^) to determine if it is necessary to review the flood risk policies, or timing of the actions, outlined in the original Plan^5^. Therefore, it is of paramount importance to have access to high-quality, multidecadal records of sea level in the Thames Estuary to accurately estimate rates of past and present changes in mean and extreme sea-level and to identify precisely when changes exceed the specific projection used in the Plan; in order that confident decisions can be made in a timely manner to move to alternative pathways if necessary.

Historical tide gauge records are an irreplaceable source of data for estimating rates of past mean sea-level rise and changes in extreme sea levels on multidecadal to century timescales^8^. Several previous studies have digitised historical tide gauge in other parts of the world. For instance: Marcos *et al*.^9–11^ captured sea level records at Cádiz, Tenerife Island, Alicante, and Santander in Spain; Talke *et al*.^12–14^ digitised historic sea level datasets for tide gauges in New York, Boston, and the Columbia Estuary (US); and Wöppelmann *et al*.^15^ digitised records at Marseille in France. However, somewhat surprisingly, there are currently relatively few long, digital tide gauge records of sea level available in the Thames Estuary. In this paper we present a new extensive sea level dataset that we have captured digitally from historical hand-written tabulated ledgers.

## Methods

In this section we start by discussing the historical context of sea level measurements in the Thames Estuary. We follow this with a description of the sea level data archive held by the Port of London Authority (PLA). Then we describe the steps we undertook to capture the tabulated datasets that were available. We then briefly describe further work that could be undertaken in the future.

### Historical context

The River Thames was the seaborne trade route to the docks of the Port of London, said to be the busiest in the world through the 19^th^ century. Safe navigation of the estuary and river at all states of the tide was thus a matter of great commercial (and military) importance. Although comprehensive records of heights and times of high tide at some of these docks were published and analysed in the early 19^th^ century^16^, there are very few surviving measurements of low tide from this early period, making accurate estimation of mean sea (or river) level before the 1820s difficult.

The removal of Old London Bridge between 1821 and 1834 led to concerns about the impact on tidal levels, and in February 1830, the UK Admiralty directed the surveyor John Augustus Lloyd to determine the difference between the mean sea level (MSL) at Sheerness and the water levels at various points on the Thames. The work that followed was to influence surveying practice for the early operations of the Ordnance Survey in the UK, and sea level measurement up to the present day. After arriving at the Admiralty dockyard in March 1830, Lloyd set up a tide gauge in the Sheerness Dock Basin which had spring loaded pointers allowing registration of the highest and lowest tide levels on a marked scale^17^. These would need to be read and reset after each tide, but could at least automatically record the high water (HW) and low water (LW) levels (if not the times) when unattended. Lloyd used the 31 feet mark on the carved stone tide scale at the dock entrance to give a tidal reference point and referred this to several bench marks he set up^18^ (e.g. http://www.bench-marks.org.uk/bm27754). He also set the zero of his tide gauge to “18 feet” above the dock entrance. This was actually 17 feet and 11 inches in the hand-written tidal register of HW and LW^19^, which was close to mean tide level (MTL) (see below). Mitchell, the master millwright at the Sheerness dockyard, who supervised the construction of Lloyds gauge, subsequently modified the gauge so that it was self-registering, recording a continuous trace of the water levels (or mareogram) on a roll of paper^19,20^, on similar principles to the gauge proposed earlier that year by the engineer to the London Dock Company^21^. Mitchell’s mechanism was operating by September 1831, and was the world’s first documented continuously recording tide gauge. Quality controlled monthly MSL data from this gauge from 1832 onwards is available from the Permanent Service for Mean Sea Level (PSMSL; https://www.psmsl.org). Similar gauges were then installed in other Admiralty Dockyards, in Plymouth, Portsmouth, and Pembroke Docks, as well as one at Bristol. Tables of twice daily HW and LW from Sheerness were also transcribed from handwritten ledgers and published^22–24^.

The second half of the 19^th^ century saw further increases in shipping volume, the embankment of much of the river, and construction of docks such as the Royal Victoria Docks in the 1850s and the Royal Albert Dock and Tilbury in the 1880s. Tidal observations in many parts of the Thames except Sheerness continued piecemeal^25,26^, without central coordination. Competition between docks and alteration of low water datums (at one point several different Admiralty Chart datums existed for different sections of the Thames) in this period has meant that any data archaeology exercise to recover unpublished 19^th^ century tide level information for the Thames estuary is challenging, despite the importance of London as a port. The situation improved after the PLA was established on 31^st^ March 1909, and thereafter proceeded to install new automatic tide gauges at various locations^27^, the archived records of which are the basis of this paper.

### Port of London Authority data archive

**Fig. 1:**
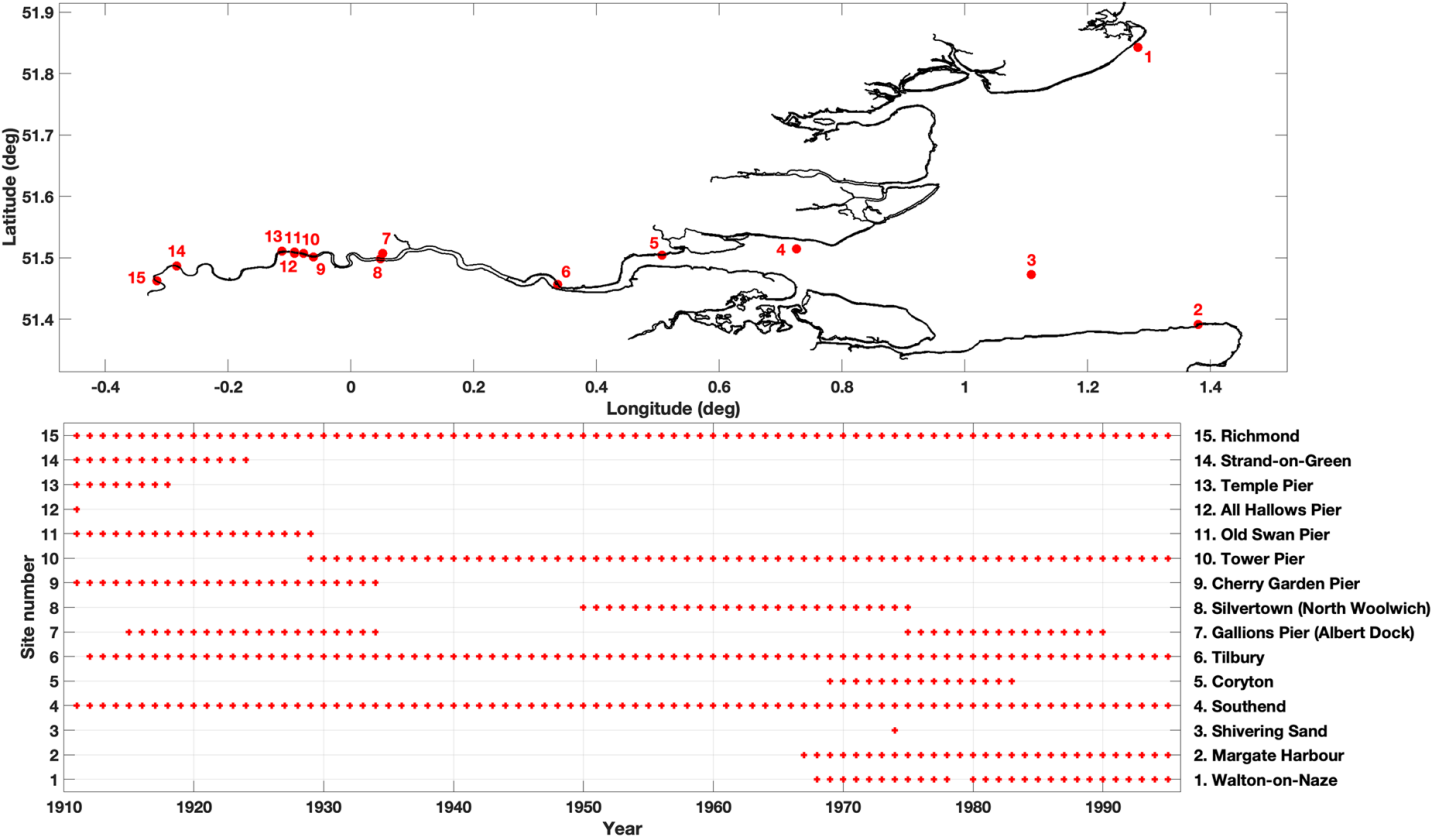
(**a**) Location of tide gauges sites and; (**b**) Duration of the datasets.

**Fig. 2:**
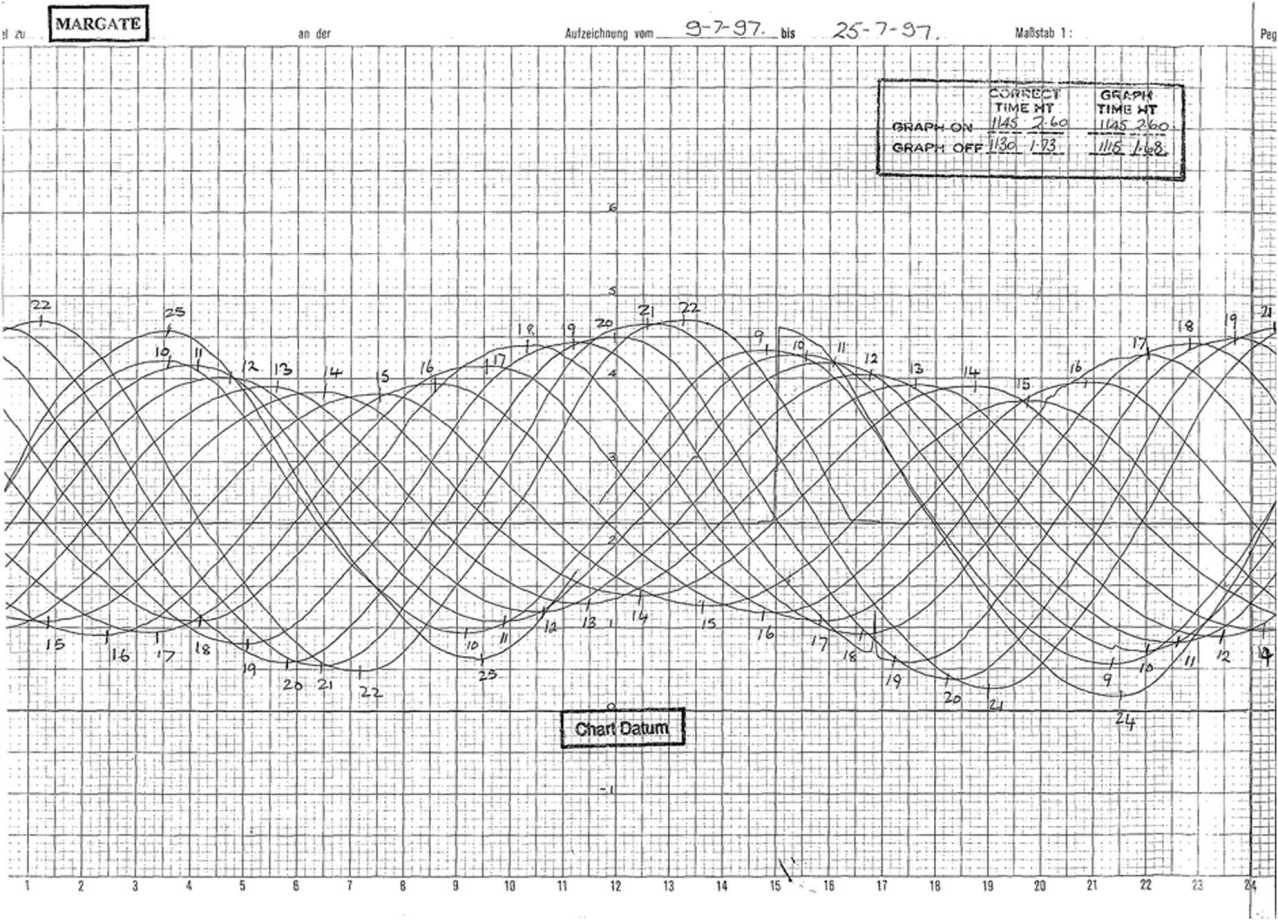
Example of a tidal chart at Margate containing 14 days of sea level measurements from 09 July 1997 to 25 July 1997.

The PLA has operated tide gauges at up to 15 sites within and adjacent to the Thames Estuary, the locations of which are shown in Fig. 1 and are listed in Table 1. The data from these tide gauges is only available digitally from 1994 onwards, or later. However, at these sites, and at other sites that are no-longer in operation, the PLA hold original tidal charts containing analogue sea level curves going back as far as 1911; an example of a tidal chart at Margate containing 14 days of sea level traces in July 1997 is shown in Fig. 2. These charts have been scanned to micro-films, held at the PLA, and the original paper charts have been destroyed. In addition to the analogue tidal charts, the PLA holds 34 books containing hand-written tabulated ledgers of twice daily values of measured HW and LW, that were read off the tidal charts at the time. The sites and periods for which these non-digital historic datasets are available are also shown in Fig. 1 and are listed in Table 1.

**Table 1:** Location and duration of the sea level datasets held by the Port of London Authority.

Gauge	Latitude (Degrees)	Longitude (Degrees)	Analogue (continuous analogue curve on microfilm)	Analogue (tabulated HW & LWs)	Digital (10 min 1994–1999, 1 min 2000 - current)
1. Walton-on-Naze	51.8428	1.2817	25/09/1967–03/11/1996	01/01/1968–31/12/1978, 01/06/1980–31/12/1995	01/01/1995 - current
2. Margate Harbour	51.3914	1.3797	21/03/1970–08/01/1997	22/09/1967–31/12/1977, 01/01/1979–31/12/1995	01/01/1994 - current
3. Shivering Sand	51.4723	1.1081	22/06/1964–09/05/1988	01/01/1974–31/12/1974	01/01/1995 – current (missing 2001)
4. Southend	51.5139	0.7255	03/01/1929–03/01/1997	01/01/1911–31/12/1995	01/01/1994 - current
5. Coryton	51.5041	0.5068	17/10/1969–23/01/1983	01/01/1969–31/12/1983	01/01/1994 - current
6. Tilbury	51.4562	0.3368	24/01/1929–12/10/1989	08/08/1912–31/12/1995	01/01/1994 - current
7. Gallions Pier (Albert Dock)	51.5069	0.0518	20/07/1950–08/05/1990	01/01/1915–07/09/1935, 01/01/1976–31/12/1990	01/01/1994 - current
8. Silvertown (North Woolwich)	51.4982	0.0478	20/07/1950–08/05/1990	20/07/1950–31/12/1975	01/01/1994 - current
9. Cherry Garden Pier	51.5013	−0.0610	—	01/01/1911–31/12/1934	—
10. Tower Pier	51.5070	−0.0775	01/01/1929–10/01/1997	23/04/1929–31/12/1995	01/01/1995 - current
11. Old Swan Pier	51.5072	−0.0919	01/01/1929–10/01/1997	01/05/1911–22/04/1929	01/01/1995 - current
12. All Hallows Pier	51.4468	0.7601	—	01/01/1911–31/01/1911	—
13. Temple Pier	51.5107	−0.1123	—	01/01/1911–31/12/1918	—
14. Strand-on-Green	51.4867	−0.2841	—	01/01/1911–31/12/1924	—
15. Richmond	51.4623	−0.3159	30/06/1962–10/01/1997	01/01/1911–31/12/1995	01/01/1995 - current

To improve understanding of historical trends in both mean and extreme sea levels throughout the Thames Estuary, it would be preferable to digitise as much of the non-digital datasets as possible. Ideally, one would wish to capture high frequency (at least hourly) measured sea level curves from the analogue tidal charts, but this is an extremely arduous and time-consuming process^28^; particularly given that the micro-films would need to first be converted to digital files. Digitising the tabulated HW and LW values, while time-consuming, is an easier task, and still provides an extremely valuable source of data. Hence, this is the dataset we capture here.

In total, there are 34 ledgers. Three of the ledgers are 35.5 cm long and 32.5 cm wide, and each sheet within contains hand written values of high and low water for all the tide gauge sites available at the time. Each page contains 4 or 5 days of data, across the available sites, for the period 1911 to July 1938. The remaining 31 ledgers are 35 cm long and between 23 and 28 cm wide. Each page of these books contains one month of data at one site, and covers the period August 1938 to the end of 1995. Across the 34 books, tabulated data is available for 15 sites (shown in Fig. 1 and listed in Table 2), for periods ranging from 1 month to 85 years. In total the equivalent of 518 years of tabulated values are available across 15 sites.

**Table 2:** The time format, units and datums for each of the historic tide gauge sites.

YEARS	Type 1: 1911–1920	Type 2: 1921–21 Aug 1934	Type 3: 22 Aug 1934-Jul 1938	Type 4: Aug 1938–1953	Type 5: 1954–1973	Type 6: 1974–1995
1. Walton-on-Naze					Ft.	Metres
					ODN	CD
					24-hour	24-hour
2. Margate					Ft.	Metres
					ODN	CD
					24-hour	24-hour
3. Shivering Sand						Metres
						CD
						24-hour
4. Southend	Ft. and In.	Ft. and In.	Ft. and In.	Ft. and In.	Ft.	Metres
	THW	THW	ODN	ODN	ODN	CD
	A.M. and P.M.	A.M. and P.M.	24-hour	24-hour	24-hour	24-hour
5. Coryton					Ft.	Metres
					ODN	CD
					24-hour	24-hour
6. Tilbury	Ft. and In.	Ft. and In.	Ft. and In.	Ft. and In.	Ft.	Metres
	THW	THW	ODN	ODN	ODN	CD
	A.M. and P.M.	A.M. and P.M.	24-hour	24-hour	24-hour	24-hour
7. Gallions Pier (Albert Dock)	Ft. and In.	Ft. and In.				Metres
	THW	THW				CD
	A.M. and P.M.	A.M. and P.M.				24-hour
8. Silvertown (North Woolwich)				Ft. and In.	Ft.	Metres
				ODN	ODN	CD
				24-hour	24-hour	24-hour
9. Cherry Garden Pier	Ft. and In.	Ft. and In.				
	THW	THW				
	A.M. and P.M.	A.M. and P.M.				
10. Tower Pier		Ft. and In.	Ft. and In.	Ft. and In.	Ft.	Metres
		THW	ODN	ODN	ODN	CD
		A.M. and P.M.	24-hour	24-hour	24-hour	24-hour
11. Old Swan Pier	Ft. and In.	Ft. and In.				
	THW	THW				
	A.M. and P.M.	A.M. and P.M.				
12. All Hallows Pier	Ft. and In.					
	THW					
	A.M. and P.M.					
13. Temple Pier	Ft. and In.					
	THW					
	A.M. and P.M.					
14. Strand-on-Green	Ft. and In.	Ft. and In.				
	THW	THW				
	A.M. and P.M.	A.M. and P.M.				
15. Richmond	Ft. and In.	Ft. and In.	Ft. and In.	Ft. and In.	Ft.	Metres
	THW	THW	ODN	ODN	ODN	ODN
	A.M. and P.M.	A.M. and P.M.	24-hour	24-hour	24-hour	24-hour

At Southend, tabulated data is available for 1911 to 1995. However, digital high frequency (at least hourly) sea level data is also available at Southend from 1929 to the end of 2020 (missing 1984, 1985, 1986, and 1987). This dataset was made available by the EA, but the original source of the data is not clear (i.e. when the data was digitised from tidal charts and by whom); although we are confident the data originates from the PLA. For this reason, at Southend, we only digitised 1911 to 1934 as for this period each digitised sheet contained the high and low water for all tide gauge sites available, thus we digitised data for all sites to keep the excel spreadsheet looking like the original ledgers, and 1983 to 1988 to capture the missing data. From the overlapping years (1929–1934, 1983, 1988), we compared the digital data with the high and low water data from the hand-written charts. We found good agreement and hence did not capture the tabulated data for the years when high frequency data is available at Southend. In total, we captured 463 years of data from 518 years of charts available across 15 tide gauge sites, for the period 1911 to 1995.

**Fig. 3:**
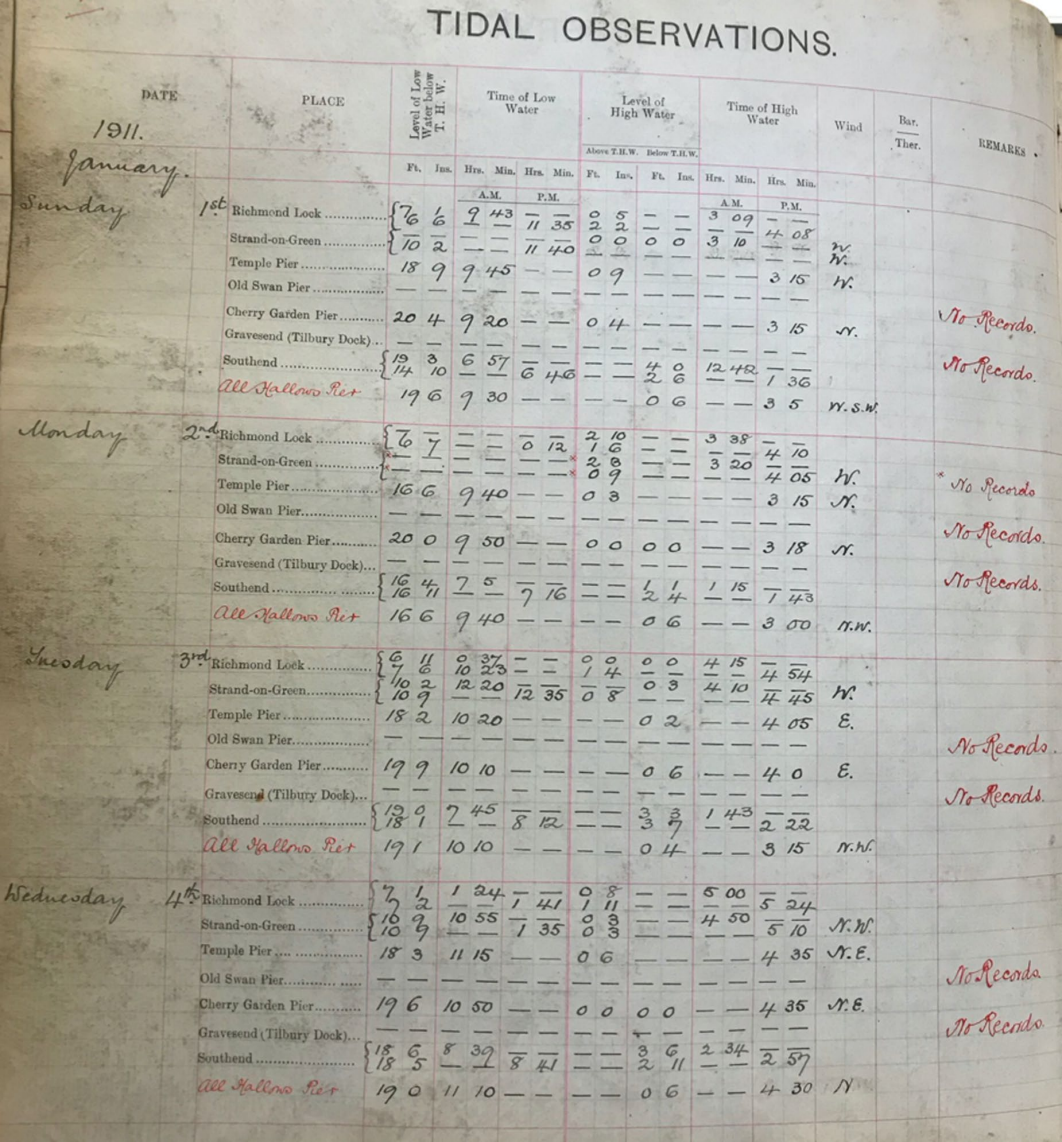
PLA hand-written tabulated ledgers, format 1.

**Fig. 4:**
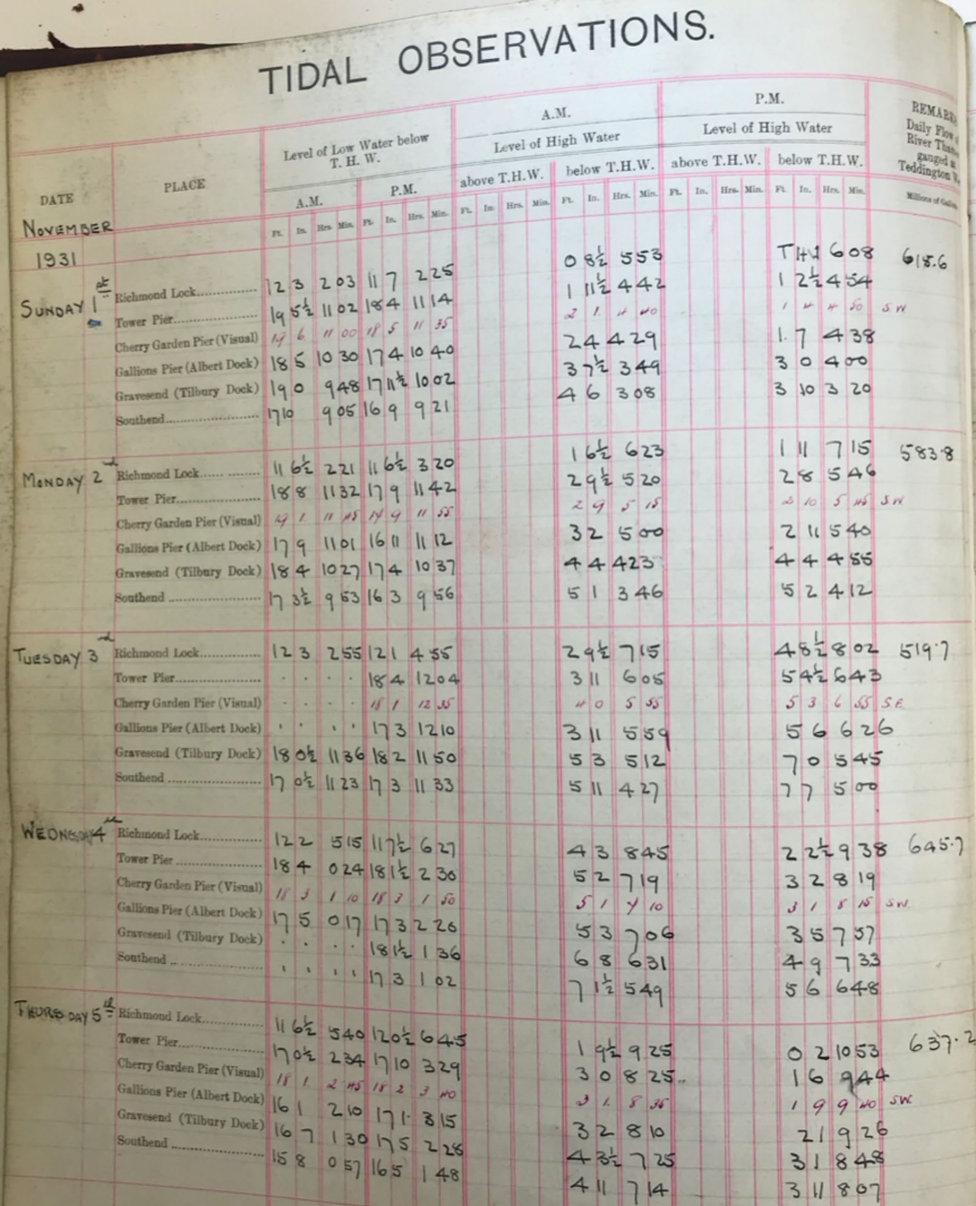
PLA hand-written tabulated ledgers, format 2.

**Fig. 5:**
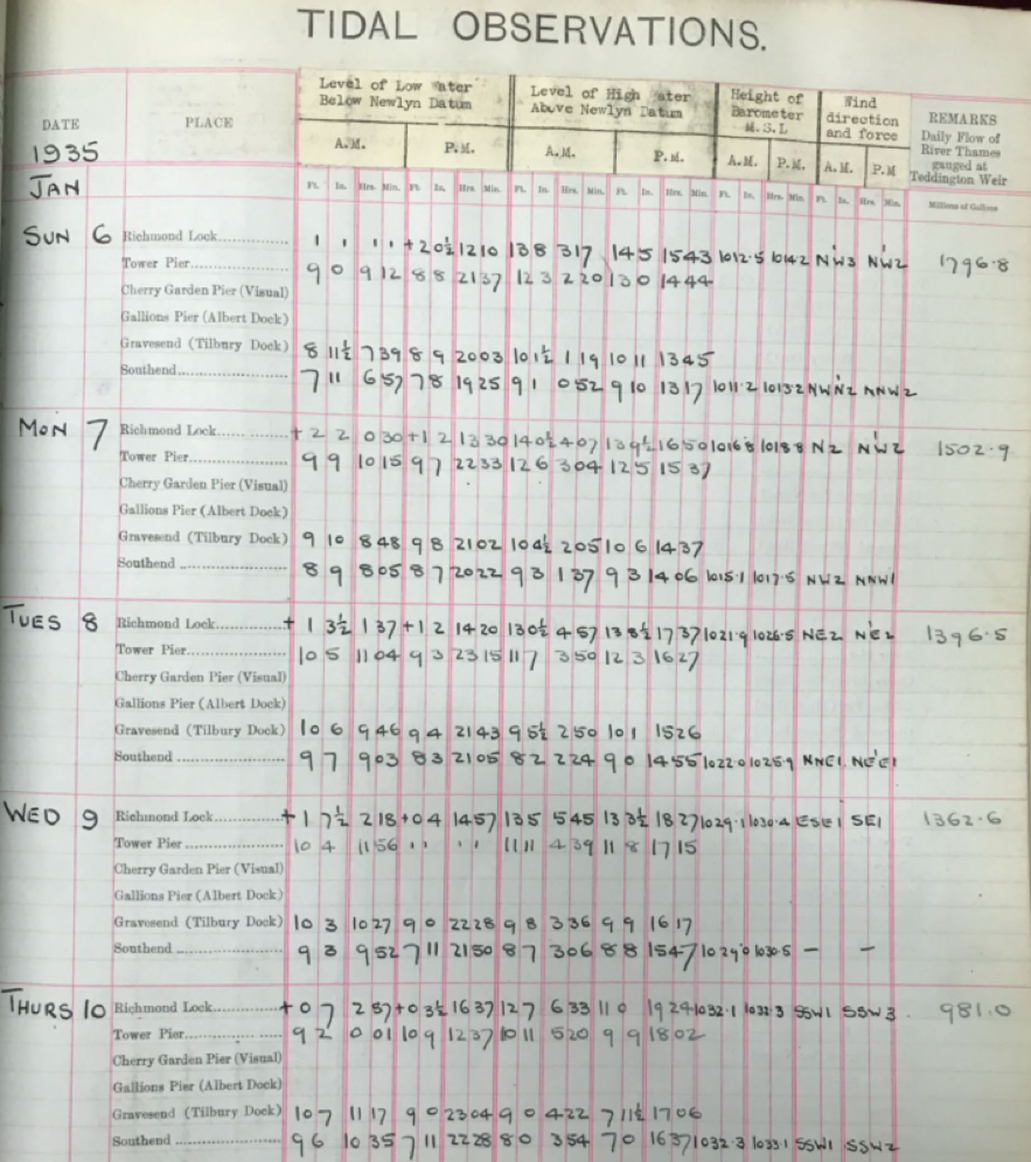
PLA hand-written tabulated ledgers, format 3.

**Fig. 6:**
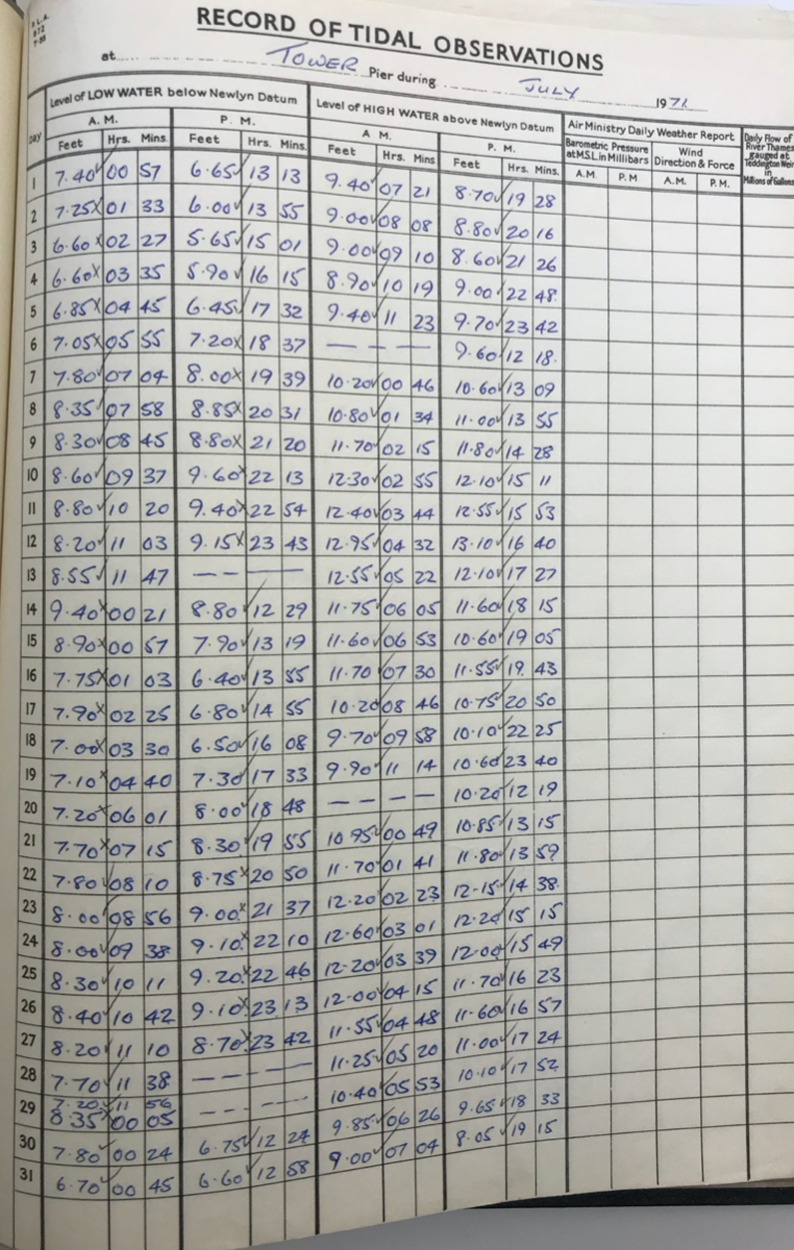
PLA hand-written tabulated ledgers, format 4.

**Fig. 7:**
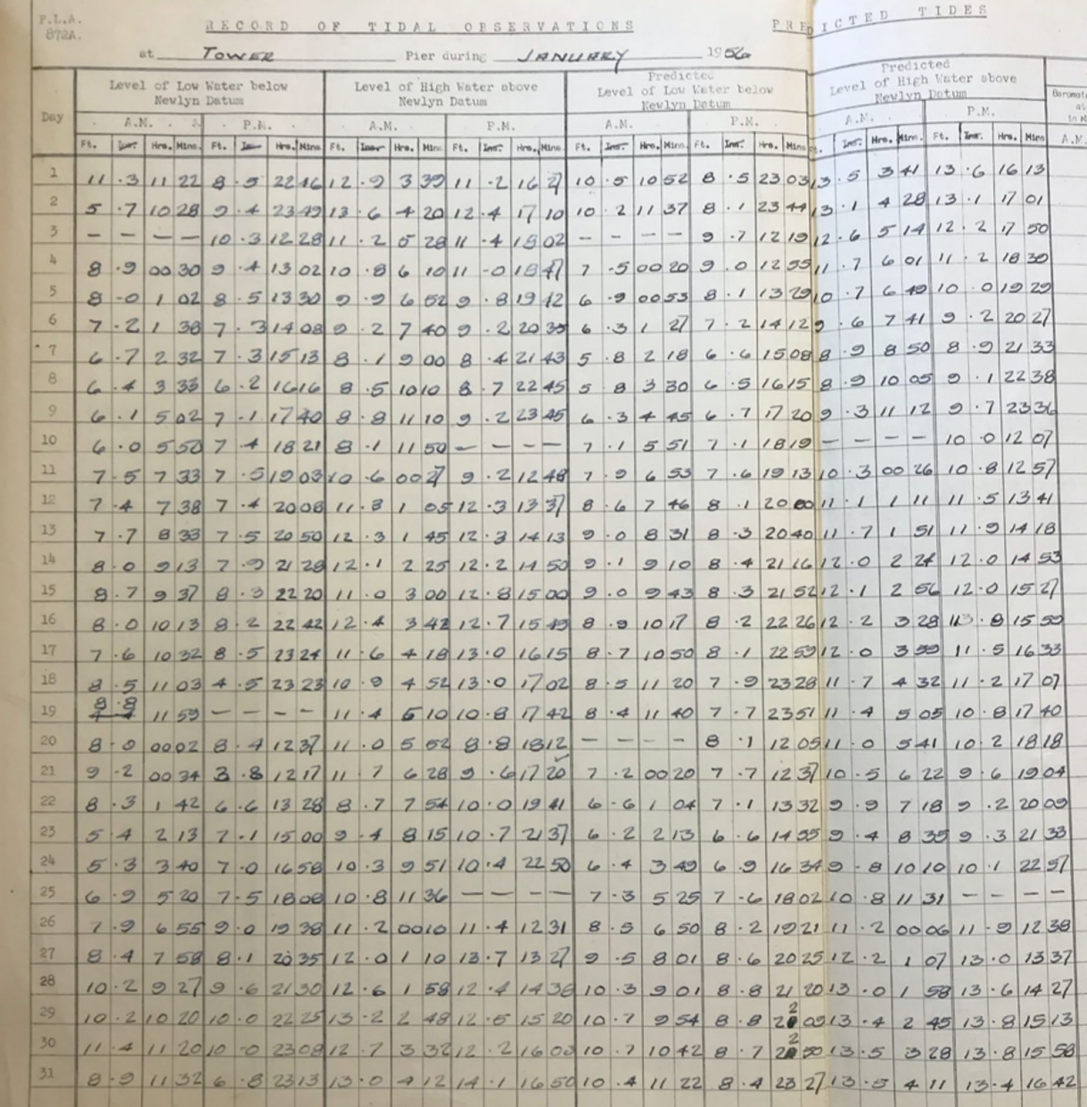
PLA hand-written tabulated ledgers, format 5.

**Fig. 8:**
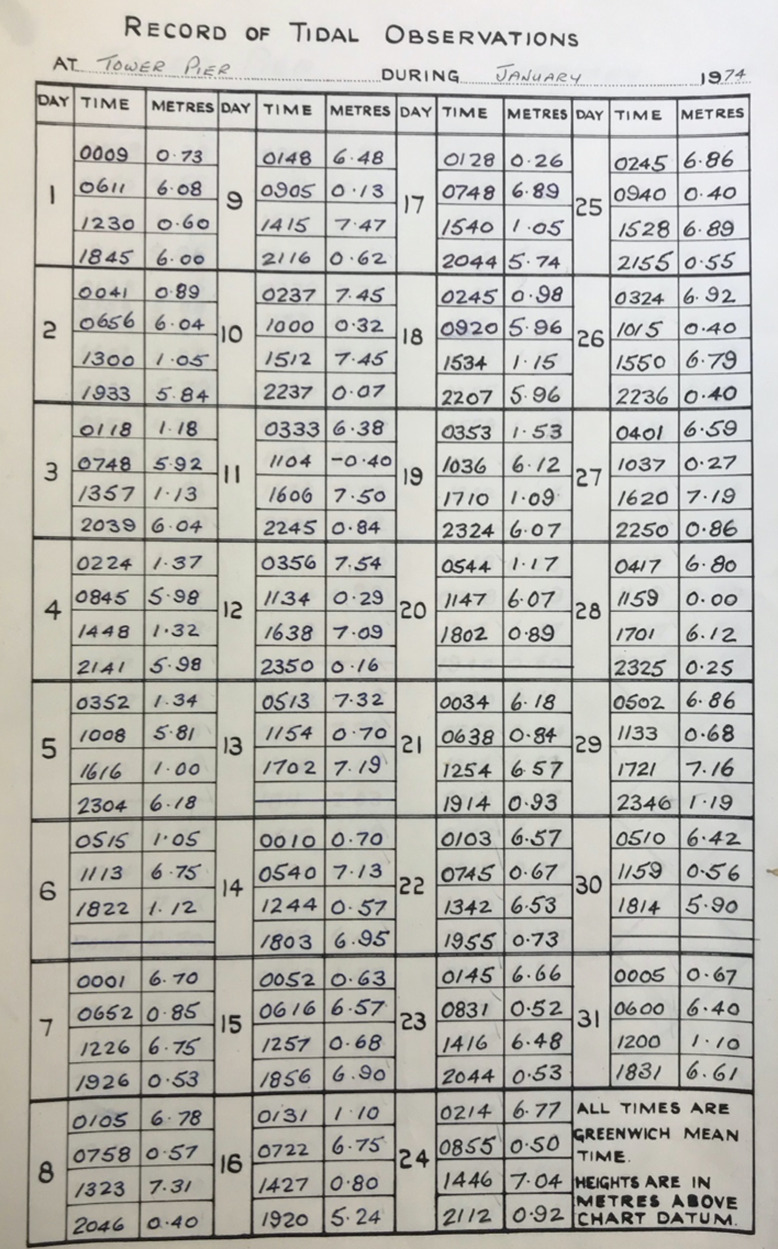
PLA hand-written tabulated ledgers, format 6.

Across the 34 ledgers, there are differences in the format of the time (12 hours or 24 hours), units of the recorded water level (feet or metres) and the reference vertical datum. We distinguished six main format types, examples of each of which are shown in Figs. 3 to 8. Types 1 to 3 are similar, and the times and heights of high water are listed for all available tide gauges sites, for the same day. In Types 4 to 6, a single page contains a whole month of high and low water values for one site.

### Data capture

The data capture and quality control stages involved four main steps, each described in turn below.

In the first step, we manually inputted the times and heights of the twice daily HW and LW values at all the available sites into Excel spreadsheets. Each Excel spreadsheet was formatted so that visually it looked similar to the format of the original ledgers. As discussed later, this made it easier to quickly input the data and check possible outlier values. On average it took 3 days to input a year’s worth of HW and LW values for the Type 1 to 3 formats. Whereas, for the Type 4 to 6 formats we were able to input about 3 years’ worth of data, per site, in one day. In total, it took us approximately 12 months to capture all the available data. The majority of the datasets were digitised by the lead author, but other authors digitised select periods.

In the second step, we wrote scripts (in the MATLAB programming language) to load in the data from across all the available spreadsheets, and output combined time-series of high and low water, for each site. To make the datasets coherent through time, corrections had to be made to convert the records into the same time format and units and make them referenced to the same vertical datum. The format of the time, units, and datum, for each of the six tabulated types, are listed in Table 2. The Type 1 and 2 tabulated sheets cover the period 1911 to 1920 and 1921 to 21 August 1934, respectively. In both these cases (Figs. 3 and 4), the time format is 12 hours, the level of water is recorded in feet and inches, and the datum is Trinity High Water (THW). The Type 3, 4 and 5 tabulated sheets cover the period 22 August 1934 to July 1938, August 1938 to 1953 and 1954 to 1973 respectively. In these three cases (Figs. 5, 6 and 7), the time format is 24 hours, the level of water is recorded in feet and inches, and the datum is Ordnance Datum Newlyn (ODN). The Type 6 tabulated sheets cover the period 1974 to 1995. In this case (Fig. 8), the time format is 24 hours, the level of water is recorded in meters, and the datum is Chart Datum (CD) at each site, except for Richmond which is recorded in ODN.

When combining time-series of HW and LW values, for each site, we first converted the date/time from the Type 1 and 2 sheets from 12-hour format to 24-hour format. We adjusted the data from Types 1 to 4 sheets, so that all water levels were relative to metres ODN. The difference between THW and ODN is 11.4 ft (3.475 m) at most sites, however, we found additional notes for Southend, Tower Pier, and Richmond indicating different values for the difference between THW and ODN for these sites (Table 3). We also converted the data from the Type 6 sheets, from CD at each site (except Richmond which already recorded in ODN) to ODN. The differences between THW and ODN or CD and ODN, are listed in Table 3 for all the sites. We also carried out specific level corrections for select sites. We found a note in one of the ledgers saying that Old Swan Pier had been relevelled on 25 June 1922 and lifted by 6 inches. Therefore, we subtracted 6 inches (0.1524 m) from all of the values before this date. We also found a note for Tilbury indicating that a levelling correction of −0.03 m should be applied for the period from 10 January 1979 to 31 December 1979, so we adjusted the levels accordingly.

**Table 3:** The corrections (in meters) used to convert all data from Trinity High Water (THW) or Chart Datum (CD) to Ordnance Datum Newlyn (ODN).

YEARS	Type 1: 1911–1920	Type 2: 1921-21 Aug 1934	Type 3: 22 Aug 1934-Jul 1938	Type 4: Aug 1938–1953	Type 5: 1954–1973	Type 6: 1974–1995
1. Walton-on-Naze					ODN	−2.16
2. Margate					ODN	−2.50
3. Shivering Sand						−2.68
4. Southend	3.3726	3.3726	ODN	ODN	ODN	−2.90
5. Coryton					ODN	−3.05
6. Tilbury	3.475	3.475	ODN	ODN	ODN	−3.12
7. Gallions Pier (Albert Dock)	3.475	3.475				−3.35
8. Silvertown (North Woolwich)				ODN	ODN	−3.35
9. Cherry Garden Pier	3.475	3.475				
10. Tower Pier		3.459	ODN	ODN	ODN	−3.2
11. Old Swan Pier	3.459	3.459				
12. All Hallows Pier	3.475					
13. Temple Pier	3.475					
14. Strand-on-Green	3.475	3.475				
15. Richmond	3.4036	3.4036	ODN	ODN	ODN	ODN

In the third step, we combined data from near-by sites, each less than 1 km from the original site, to produce more continuous records of longer data length. The tide gauge was removed from Old Swan Pier and installed at Tower Pier on 23 February 1929, so we combined the records from Old Swan Pier and Tower Pier. We call this combined dataset London Bridge. We also combined the records from Gallions and North Woolwich, given their close vicinity, and called this combined dataset Silvertown, as it is near the current Silvertown PLA tide gauge.

In the fourth step, we extracted twice daily HW and LW value from the digital high frequency sea level dataset at Southend available from 1929 to 1983 and 1987 to the end of 1995. The extracted high and low water values from this digital dataset where then combined with the historic dataset captured from the ledgers at Southend.

At Southend, Tilbury, Silvertown, London Bridge and Richmond, this new dataset results in a near continuous record of HW and LW from 1911 to 1995. When these historic datasets are combined with digital records available from the PLA since 1995, the sea level time-series span the 111-year period from 1911 to 2021, and are now amongst the longest sea level records available for the UK.

### Future work

At some point in the future we would like to digitise the original tidal charts containing the analogue sea level curves.

## Data Records

This new digitised sea level dataset is freely available to the public through an unrestricted repository archived with the British Oceanographic Data Centre (BODC)^29^, and is formatted according to their international standards. The dataset consists of three files. The first file is a zip file containing photographs (in HEIC and jpg format) of every tabulated sheet from the 34 ledgers. The second file is a zip file containing Excel spreadsheets (XLSX), designed to look similar to the 6 different ledgers formats. These contain the raw digitised tabulated dataset of HW and LW values. Prior to 1935, each Excel file contains one year of data for all the sites. From 1935 onwards, each Excel file contains a year of data for a specific site. Each separate sheet within these Excel files contains the tabulated data for any given month. Note, we have also provided the data in CSV (comma separate variable) files for those with no access to Excel. The third file is a zip file contain the final datasets for each site in text files containing the combined, corrected and quality-controlled time-series of high and low water at the 13 sites. Each of the files is self-describing and accompanied by metadata.

## Technical Validation

It was not possible for us to check that every single HW and LW value was inputted correctly, because of the great length of time it would take to do this manually. However, we designed the data capture processes, and subsequent quality control, to ensure the records were captured as accurately as possible. As previously discussed, each Excel spreadsheet was formatted so that it looked visually similar to the format of the original ledgers to make it easier to quickly input the data and check for possible outlier values later. After completing the digitisation processes, we wrote MATLAB scripts to load in the data from across all of the available spreadsheets, correcting for differences in time, unit format and datums, and output combined time-series of HW and LW, for each site. We then plotted the HW and LW time-series for each site. We visually inspected the dataset for each site and identified values that we considered lay outside of a reasonable expected range. We then manually checked each of these values against the original ledgers. In most cases the spurious values arose as a result of us inputting the data with the decimal in the wrong place. Once we had checked each site, we ran the MATLAB scripts again, and undertook another round of checks. We continued until we were confident no outliers remained. We have deliberately archived the Excel spreadsheets containing the raw values, for each year and site, before applying any time, unit or datum corrections, so that any particular value can be checked against the original values in the ledgers at any time in the future. Furthermore, we have photographed and archived each tabulated sheet to aid this process.

## Data Availability

The programming scripts written (in MATLAB) to load in the data from across all the available spreadsheets, and output combined time-series of high and low water, for each site, are available from https://github.com/ivanhaigh/Thames-Sea-Level-Data.
